# Cardiovascular Disease Markers in Schizophrenia During Negative Symptoms and Remission Periods

**DOI:** 10.3390/jcm14072288

**Published:** 2025-03-27

**Authors:** Okan Imre, Gurkan Imre, Mehmet Mustu, Omer Acat, Rahim Kocabas

**Affiliations:** 1 Department of Psychiatry, Faculty of Medicine, Karamanoglu Mehmetbey University, Karaman 70200, Turkey; 2 Department of Cardiology, Faculty of Medicine, Bilecik Seyh Edebali University, Bilecik 11200, Turkey; gurkan.imre@bilecik.edu.tr; 3 Department of Cardiology, Faculty of Medicine, Karamanoglu Mehmetbey University, Karaman 70200, Turkey; drmustumehmet@kmu.edu.tr; 4 Department of Public Health, Faculty of Medicine, Karamanoglu Mehmetbey University, Karaman 70200, Turkey; omeracat@kmu.edu.tr; 5 Department of Biochemistry, Faculty of Medicine, Karamanoglu Mehmetbey University, Karaman 70200, Turkey; rkocabas@kmu.edu.tr

**Keywords:** schizophrenia, cardiac diseases, biomarkers

## Abstract

**Objectives**: This study aims to investigate cardiovascular disease markers in patients with schizophrenia and to contribute to the early indication of asymptomatic cardiovascular diseases in these patients. In our study, there are three groups: schizophrenia with negative symptoms (SCH-N), schizophrenia in remission (SCH-R), and a healthy control group (HC). In these groups, there were compared parameters such as lipid panel, Atherogenic Index (AIP), Triglyceride-glucose (TyG) index, Castelli Risk Index-1 (CRI-I), Castelli Risk Index-2 (CRI-II), and Atherogenic Coefficient (AC), which are associated with the risk of cardiovascular disease. **Methods:** The participants of the study were from the HC group and schizophrenia patients aged between 18 and 65 who were followed up at the Psychiatry Clinic of Karaman Hospital. This cross-sectional case–control study consists of the SCH-N (n:20), the SCH-R (n:23), and the HC (n:21) groups. Those with cardiovascular, endocrine, and inflammatory diseases, those with alcohol and substance addiction, those using drugs other than psychiatric drugs, and those lacking informed consent were excluded from the study. Patients in active psychotic episodes were also excluded from the study due to communication difficulties. All data were analyzed using SPSS 25.0 package program in a computer environment. The conformity of continuous data to normal distribution was evaluated with normality test value, q-q plot, skewness, and kurtosis. For significant results in the ANOVA test, pairwise comparisons were conducted using the post hoc Bonferroni correction when variances were homogeneously distributed. Similarly, for significant results in the Kruskal–Wallis Test, pairwise comparisons were performed using the Dunn–Bonferroni test. In this study, values less than *p* < 0.05 were considered statistically significant. **Results:** When all groups were compared, the increase in the TGs, TyG index, AIP, CRI-I, CRI-II, and AC values in the SCH-R group compared to the HC group was found to be statistically significant (*p* < 0.001, *p* < 0.001, *p* < 0.001, *p* < 0.001, *p* = 0.015, *p* < 0.001; sequentially). **Conclusions:** This study revealed that cardiovascular risk markers in schizophrenia patients showed significant differences. In particular, the elevation in parameters such as TGs, TyG index, AIP, CRI-I, CRI-II, and AC indicates that schizophrenia patients have an increased risk for cardiovascular diseases. Therefore, it is recommended that schizophrenia patients be closely monitored for cardiovascular risk factors and to intervene early.

## 1. Introduction

Schizophrenia is a serious, lifelong neuropsychiatric disorder that typically begins in late adolescence. The lifetime prevalence of schizophrenia is thought to be approximately 0.7% [1]. Schizophrenia is one of the top 20 causes of disability worldwide [2]. This disease affects the patient’s biopsychosocial with negative symptoms, positive symptoms, and disorganized behaviors [3]. People with schizophrenia typically die 15 years earlier than the general population, with cardiovascular disease being one of the leading causes of death [4]. According to a meta-analysis of data, people with schizophrenia are at increased risk for coronary heart disease and congestive heart failure due to many factors, including side effects of medications, metabolic syndrome, and smoking [5]. In addition, inactivity and social withdrawal, which are among the negative symptoms of schizophrenia, can also increase the risk of cardiovascular disease. Although some studies have excluded conditions that increase the risk of cardiovascular disease, such as medications, diet, and inactivity, it has been stated that the risk of cardiovascular disease in schizophrenia is high due to genetic reasons [6]. Therefore, monitoring cardiovascular disease in schizophrenia is important. Early indication of asymptomatic cardiovascular diseases in patients with schizophrenia will both increase the survival of patients and improve their quality of living. Recently, some studies have identified various predictive risk factors for cardiovascular diseases, such as Atherogenic Index (AIP), Triglyceride-glucose (TyG) index, Castelli Risk Index-1 (CRI-I), Castelli Risk Index-2 (CRI-II), and Atherogenic Coefficient (AC) [7,8]. Therefore, in order to evaluate these early markers in patients with schizophrenia, we compared TyG index, AIP, CRI-I, CRI-II, and AC markers among the SCH-N, the SCH-R, and the HC groups.

## 2. Materials and Methods

### 2.1. Ethical Approval and Participants

In this cross-sectional case–control study, the patient group (SCH-N and SCH-R) consisted of schizophrenia patients aged between 18 and 65 who were followed up at the Karaman Hospital Psychiatry Clinic, and all patients met the criteria for schizophrenia (according to DSM-5-TR) [9]. The study was approved by the Karamanoğlu Mehmetbey University Faculty of Medicine Ethics Committee (Date: 5 September 2024, No: 09-2024/06).

### 2.2. Exclusion Criteria

Those with cardiovascular, endocrine, and inflammatory diseases, those with alcohol and substance addiction, those using drugs other than psychiatric drugs, and those lacking informed consent were excluded from the study. Patients in active psychotic episodes were also excluded from the study due to communication difficulties. The severity of symptoms and negative and remission criteria in schizophrenia patients were measured by a psychiatrist using the Positive and Negative Syndrome Scale (PANSS). The absence of symptoms for at least 6 months was considered as the patient being in remission [10,11]. There were 43 patients with schizophrenia in the study, 20 of whom had negative symptoms and 23 who were in remission.

In the HC group, those with any history of psychiatric illness and those who met the exclusion criteria mentioned above were excluded from the study. First, the study procedure was explained to all participants, and then written informed consent was obtained from all participants. Informed consent was obtained from parents of schizophrenia patients who were unable to give written informed consent. This study was conducted according to the revised version (October 2013) of the Declaration of Helsinki [12].

They recorded the sociodemographic data of schizophrenia patients. All blood specimens were taken from the participants after 8 h of fasting. For serum samples, blood samples taken from participants were first allowed to clot for 30 min. Then, the samples were centrifuged at 1800× *g* for 10 min at 8 °C. After obtaining the serum, routine biochemical tests such as total cholesterol (TC), high-density lipoprotein cholesterol (HDLc), low-density lipoprotein cholesterol (LDLc), triglyceride (TGs), and glucose were performed immediately. Commercial kits suitable for the tests were used on the Beckman Coulter AU5800 auto-analyzer to study the test samples (Beckman Coulter, Indianapolis, IN, USA). Cardiovascular markers were calculated from the biochemical data with the following formulas:

Triglyceride-Glucose Index (TyG): log (TGs × Fasting glucose)/2

Atherogenic Index (AIP): log (TGs/HDLc)

Castelli Risk Index-1 (CRI-I): TC/HDLc

Castelli Risk Index-2 (CRI-II): LDLc/HDLc

Atherogenic Coefficient (AC): (TC-HDLc)/HDLc

### 2.3. Positive and Negative Syndrome Scale (PANSS)

The PANSS method was developed by Kay and colleagues. This scale has three subcategories: positive, negative, and general psychopathology. A trained expert measures the severity of the disease by rating 30 different symptoms on this scale from 1 to 7. This scale, which is a clinical interview, takes approximately 40–50 min to complete. The minimum score on the PANSS is 30 and the maximum is 210. A higher score indicates a more severe disease. If the positive-scale score is higher than the negative-scale score and shifts to the positive end, it is considered as positive-symptom schizophrenia. If the positive-scale score is lower than the negative-scale score and the score obtained when the positive score is subtracted from the negative score shifts to the negative end, it is considered as negative-symptom schizophrenia [13].

### 2.4. Statistical Analysis

All data were analyzed using SPSS 25.0 package program in a computer environment. The conformity of continuous data to normal distribution was evaluated with normality test value, q-q plot, skewness, and kurtosis. In the analysis of continuously distributed variables following a normal distribution, an independent samples *t*-test was used to determine differences between two independent groups, while a one-way ANOVA was applied for comparisons involving more than two independent groups. In cases where the data did not follow a normal distribution for more than two independent groups, the Kruskal–Wallis test was employed. For significant results in the ANOVA test, pairwise comparisons were conducted using the post hoc Bonferroni correction when variances were homogeneously distributed. Similarly, for significant results in the Kruskal–Wallis test, pairwise comparisons were performed using the Dunn–Bonferroni test. In this study, values less than *p* < 0.05 were considered statistically significant.

## 3. Results

When the SCH-N, SCH-R, and HC groups were compared in terms of sociodemographic data, there was no difference between the groups in terms of gender, body mass index, age of onset of disease, and depot antipsychotic, antidepressant, and mood stabilizer medication use. There was a difference between the groups according to mean age, and the mean age of SCH-N was higher than that of the HC group (*p* = 0.002). Compared to the SCH-N group, the use of Olanzapine and Clozapine in the SCH-R group was found to be significantly higher (*p* = 0.006), (Table 1).

**Figure 1:**
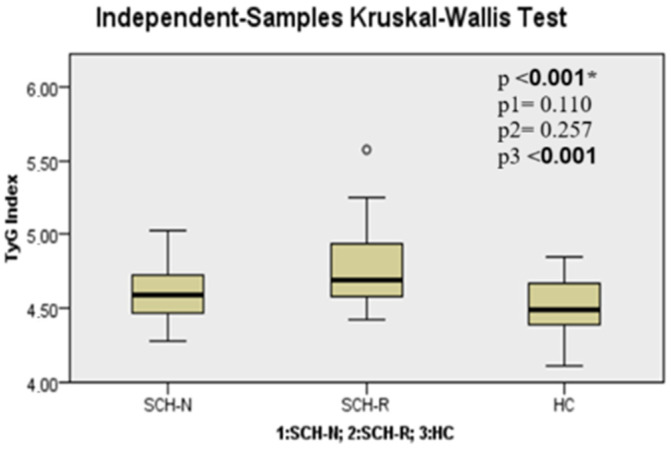
Triglyceride-glucose index level among groups. Note: * Kruskal–Wallis test, P1: SCH-N vs. SCH-R, P2: SCH-N vs. HC, P3: SCH-R vs. HC. SCH-N: schizophrenia with negative symptoms, SCH-R: schizophrenia in remission, HC: healthy control.

**Figure 2:**
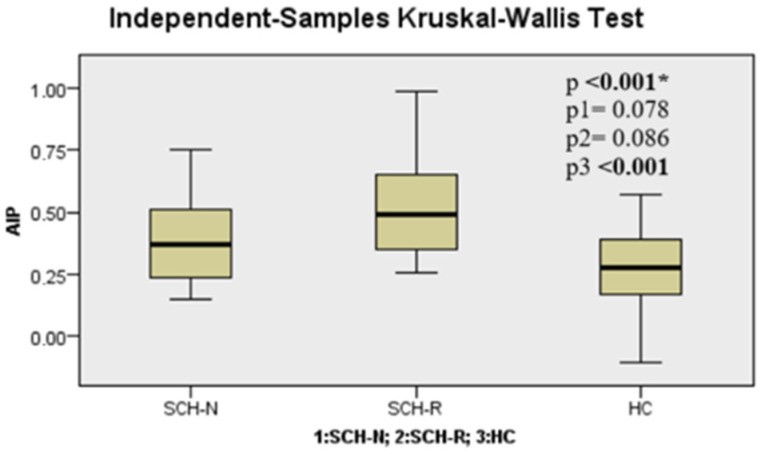
Atherogenic index level among groups. Note: * Kruskal–Wallis test, P1: SCH-N vs. SCH-R, P2: SCH-N vs. HC, P3: SCH-R vs. HC. SCH-N: schizophrenia with negative symptoms, SCH-R: schizophrenia in remission, HC: healthy control.

**Figure 3:**
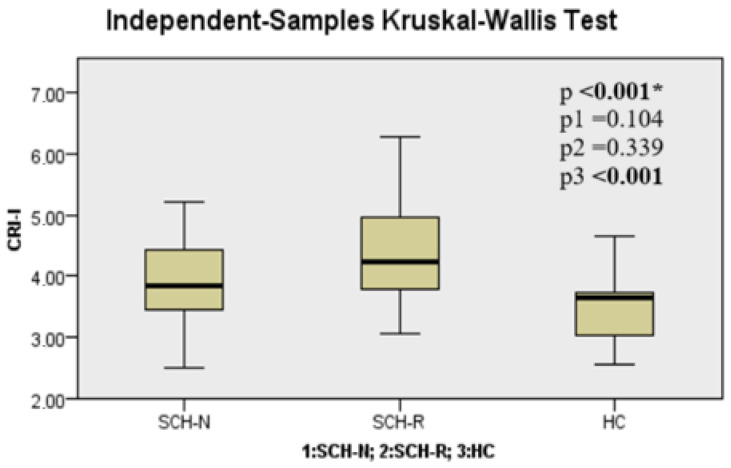
Castelli Risk Index-1 level among groups. Note: * Kruskal–Wallis test, P1: SCH-N vs. SCH-R, P2: SCH-N vs. HC, P3: SCH-R vs. HC. SCH-N: schizophrenia with negative symptoms, SCH-R: schizophrenia in remission, HC: healthy control.

**Figure 4:**
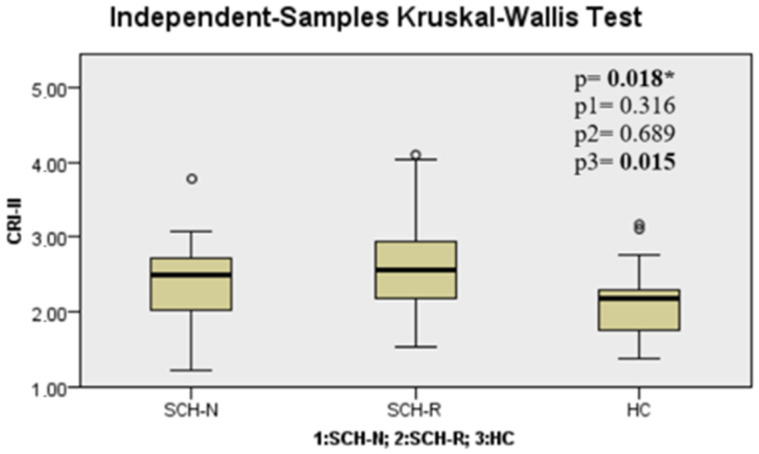
Castelli Risk Index-II level among groups. Note: * Kruskal–Wallis test, P1: SCH-N vs. SCH-R, P2: SCH-N vs. HC, P3: SCH-R vs. HC. SCH-N: schizophrenia with negative symptoms, SCH-R: schizophrenia in remission, HC: healthy control.

When the SCH-N, SCH-R, and HC groups were compared in terms of TGs, TyG index, AIP, CRI-I, CRI-II, and AC parameters, it was seen that there was a significant difference between the groups (*p* < 0.001, *p* < 0.001, *p* < 0.001, *p* < 0.001, *p* = 0.018, and *p* < 0.001, respectively) (Table 2). These differences were found to be significantly higher in the SCH-R than in HC for TGs (*p* < 0.001). TyG index, AIP, CRI-I, CRI-II, and AC parameters were found to be significantly higher in the SCH-R group than in HC (*p* < 0.001, *p* < 0.001, *p* < 0.001, *p* = 0.015, and *p* < 0.001, respectively) (Figure 1, Figure 2, Figure 3 and Figure 4, Table 2).

## 4. Discussion

In this study, biochemical markers related to cardiovascular diseases were compared among the SCH-N, SCH-R, and HC groups. Our results show that schizophrenia patients in remission have a high risk of cardiovascular diseases.

In our study, no significant difference was found between the fasting glucose levels between the groups. We think that this is because we excluded patients with metabolic syndrome and diabetes in the study. Serum TC, LDLc, TGs levels, and TyG index were found to be higher in both SCH-N and SCH-R groups compared to the HC group. However, only TGs levels and TyG index were found to be significantly higher in the SCH-R group compared to the HC group. The reason why these parameters were significantly higher in the SCH-R (91.3%) group but not in the SCH-N group may be due to the lower use of Olanzapine and Clozapine in the SCH-N group (55%) (Table 1 and Table 2). The high levels of TC, LDLc, TGs, and TyG in schizophrenia patients are consistent with the literature. Meta-analysis studies show that dyslipidemia is common in schizophrenia patients and that this is related to both the nature of the disease and the antipsychotic drugs used [14,15]. In addition, a study on untreated schizophrenia patients reported that approximately 20% of schizophrenia patients had insulin resistance and dyslipidemia regardless of treatment [16,17]. Chronic stress in schizophrenia may cause increased cortisol levels, disruption of the oxidant–antioxidant balance, and, in the future, insulin resistance and neuronal insulin resistance. In addition, decreased physical activity and poor eating habits may also increase fat storage, ultimately contributing to the development of dyslipidemia and this negative progression [18,19].

The fact that TG levels and TyG Index were found to be significantly higher only in the SCH-R group may be related to the higher use of second-generation antipsychotics Olanzapine and Clozapine in the SCH-R group (91.3%). As is known, these two antipsychotics increase TG levels more than other antipsychotics [20,21]. TG levels and TyG Index are known to be predictors of cardiovascular disease [22,23]. TGs can directly impair blood flow and increase intravascular coagulation. It can lead to endothelial dysfunction. It contributes to the formation of atherosclerosis by forming plaques in the vascular walls. It can indirectly increase the risk of cardiovascular disease by mediating the formation of insulin resistance and type 2 diabetes and metabolic syndrome [24,25]. In the literature, low HDLc levels have been reported in schizophrenia patients both before and after treatment [26]. Low HDLc levels may increase the risk of cardiovascular disease by affecting inflammatory and oxidative processes [27]. In our study, HDLc levels were found to be lower in the SCH-R group, consistent with the literature. However, it was not statistically significant. The lower HDLc levels may be due to the higher use of Olanzapine and Clozapine [28]. In addition, the lack of significant decrease in HDLc may be due to individual variables such as diet, exercise level, and genetic factors [29]. In this study, HDLc level was almost the same in the SCH-N group compared to the HC group. These data are inconsistent with most of the data in the literature. However, one study found that HDLc levels were higher in women with SCH-N [30]. The difference in the few studies that are not compatible with the literature may be due to the small sample group, the use of antipsychotics with few metabolic side effects, and individual differences such as diet and exercise. Studies with a large sample including dietary habits, exercise, genetic factors, and other antipsychotic drugs may yield more accurate results. Increased triglyceride and LDLc and decreased HDLc are known to lead to atherogenic dyslipidemia and predispose to cardiovascular disease before the onset of metabolic syndrome. These parameters have been identified as important markers of cardiovascular disease [31]. However, rather than evaluating these lipid parameters individually, it has been stated that serum lipid ratios are better at predicting cardiovascular disease, and lipid ratios can predict cardiovascular disease even if lipid values are within the normal range [32]. Therefore, atherogenic indices such as AIP, CRI-I, CRI-II, and AC have started to be used as predictors of cardiovascular disease in the asymptomatic early period [33].

In our study, atherogenic indices such as AIP, CRI-I, CRI-II, and AC were found to be higher in the SCH-N and SCH-R groups compared to the HC group. However, there was a statistically significant difference only between the SCH-R and HC groups (*p* < 0.001, *p* < 0.001, *p* = 0.015, *p* < 0.001). This may be due to the higher use of Olanzapine and Clozapine in the SCH-R (91%) group compared to other antipsychotics. These findings are consistent with previous studies emphasizing the increased risk of cardiovascular disease in patients with schizophrenia [34]. In addition, as mentioned in the paragraph above, the reason why there was no significant difference in the SCH-N group may be due to the lower rate (55%) of Olanzapine and Clozapine use in the SCH-N group compared to other antipsychotics.

The AIP index is associated with lipoprotein particle size and the development of atherosclerosis. High AIP levels have been associated with small and dense LDL particles, increasing the risk of atherosclerosis. Studies suggest that AIP may be a stronger marker than other lipid indicators in predicting cardiovascular disease [35,36,37]. There are a few studies in which atherogenic indices such as AIP, CRI-I, CRI-II, and AC were studied together in the SCH-N and SCH-R group [38]. In a study conducted by Onen et al. [38], AIP, CRI-I, CRI-II, and AC levels were found to be significantly higher in the SCH group using antipsychotics. In the same study, AIP, CRI-I, CRI-II, and AC were found to be significantly higher in the SCH group that did not use medication compared to HC. This finding indicates that atherogenic indices were high in the SCH group regardless of whether they received treatment or not and that these indices increased even more with medication use. It is concluded that atherogenic indices do not only change due to medication use but, also, conditions such as not paying attention to diet, inactivity, and stress can lead to this situation [38]. As a result, schizophrenia patients are at risk for cardiovascular disease even if they are in remission. This situation may be due to reasons such as inactivity, side effects of drugs used, poor nutritional status, and inattention to health. Therefore, the relevant psychiatrist should not neglect the risk of cardiovascular disease. A cardiologist should be consulted at certain periods. If this situation is neglected, it may result in a decrease in the quality of life, disabilities, and early death.

### Limitations

The lack of evaluation of other antipsychotic drugs, depot antipsychotic types, duration of pharmacological treatment, exercise, lifestyle, nutritional status, family burden, and social support status should be considered as a limitation. The sample size of the study was relatively small and did not include positive symptomatic schizophrenia patients. Stronger results could be obtained with a larger sample. Olanzapine and Clozapine were not used equally compared to other antipsychotics in the groups. This study was cross-sectional; more meaningful results can be obtained with larger studies that include a larger number of patients and evaluate all these limitations

## 5. Conclusions

This study supports the fact that schizophrenia is associated with metabolic and cardiovascular risk factors in patients with schizophrenia with negative symptoms and in remission without developing metabolic syndrome. It is recommended that schizophrenia patients be closely monitored for metabolic and cardiovascular disease risk and be addressed with a multidisciplinary approach.

## Figures and Tables

**Table 1:** Comparison of sociodemographic data between the schizophrenia groups and the healthy control groups.

	**SCH-N (n: 20)** **Median (Q1–Q3)** **Mean ± SD**	**SCH-R (n: 23)** **Median (Q1–Q3)** **Mean ± SD**	**HC (n: 21)** **Median (Q1–Q3)** **Mean ± SD**	*p*	P1	P2	P3
Age	51.25 ± 8.65	44.83 ± 10.20	38.29 ± 14.34	0.007 *	0.205	**0.002**	0.181
(Male) n (%)	17 (85.0%)	16 (69.6%)	19 (90.5%)	0.204 ^€^			
(Female) n (%)	3 (15.0%)	7 (30.4%)	2 (9.5%)				
BMI	27.90 (24.3–30.08)	28.0 (24.5–32.0)	27.5 (24.05–31.20)	0.874 ^β^			
Olanzapine Or Clozapine/Other antipsychotics	11(55%)	21(91.3%)		**0.006 ^γ^**			
**Age of onset of disease**	23.68 ± 6.35	25.78 ± 10.05		0.435 *			
**illness duration**	27.70 ± 9.14	18.17 ± 8.56		0.001 *			
**Depot antipsychotic n (%)**	7 (35.0%)	10 (43.5%)		0.571 ^γ^			
**Antidepressant n (%)**	9 (45.0%)	12 (52.2%)		0.639 ^γ^			
**Mood stabilizer n (%)**	5 (25.0%)	6 (26.1%)		0.935 ^γ^			

Note: * independent samples *t*-test, ^β^: Kruskal–Wallis test, ^γ^: chi square, ^€^: Fischer’s exact test. P1: SCH-N vs. SCH-R, P2: SCH-N vs. HC, P3: SCH-R vs. HC. SCH-N: schizophrenia with negative symptoms, SCH-R: schizophrenia in remission, HC: healthy control.

**Table 2:** Comparison of biochemical parameters between schizophrenia groups and healthy control groups.

	**SCH-N (n: 20)** **Median (Q1–Q3)** **Mean ± SD**	**SCH-R (n: 23)** **Median (Q1–Q3)** **Mean ± SD**	**HC(n: 21)** **Median (Q1–Q3)** **Mean ± SD**	***p***	**P1**	**P2**	**P3**
Glucose (mg/dL)	83.5 (77.5–93.5)	89.0 (84.0–101.0)	92.0 (83.0–100.5)	0.069 ^β^			
TC (mg/dL)	190.0 (158.5–234.8)	200.0 (170.0–208.0)	171.0 (139.0–190.0)	0.075 ^β^			
HDLc (mg/dL)	50.30 ± 11.20	45.65 ± 7.86	49.10 ± 10.38	0.276 *			
LDLc (mg/dL)	117.01 ± 34.03	120.76 ± 33.3	104.09 ± 31.44	0.227 *			
Triglyceride (mg/dL)	118.0 (92.3–148.3)	143.0 (117.0–177.0)	85.0 (65.5–120.5)	**<** **0.001 ^β^**	0.309	0.069	**<** **0.001**
TyG Index	4.62 ± 0.21	4.78 ± 0.29	4.49 ± 0.21	**<** **0** **.** 001 *	0.110	0.257	**<** **0.001**
AIP	0.39 ± 0.18	0.53 ± 0.22	0.26 ± 0.19	**<** **0** **.** 001 *	0.078	0.086	**<** **0.001**
CRI-I	3.90 ± 0.74	4.40 ± 0.90	3.52 ± 0.58	**<** **0** **.** 001 *	0.104	0.339	**<** **0.001**
CRI-II	2.36 ± 0.62	2.67 ± 0.71	2.13 ± 0.49	**0.018** *	0.316	0.689	**0.015**
AC	2.90 ± 0.74	3.40 ± 0.90	2.52 ± 0.58	**<** **0** **.** 001 *	0.104	0.339	**<0.001**

Note: * one-way ANOVA test, ^β^: Kruskal–Wallis test, P1: SCH-N vs. SCH-R, P2: SCH-N vs. HC, P3: SCH-R vs. HC. SCH-N: schizophrenia with negative symptoms, SCH-R: schizophrenia in remission, HC: healthy control. TC: total cholesterol, HDLc: high-density lipoprotein cholesterol, LDLc: low-density lipoprotein cholesterol, TyG index: Triglyceride-glucose index, AIP: Atherogenic index, CRI-I: Castelli Risk Index-1, CRI-II: Castelli Risk Index-2, AC: Atherogenic Coefficient.

## Data Availability

The data used to support the research findings are available upon request from the corresponding author. The data are not publicly available due to ethical restrictions.
